# Does the Hearing Sensitivity in Thorny Catfishes Depend on Swim Bladder Morphology?

**DOI:** 10.1371/journal.pone.0067049

**Published:** 2013-06-25

**Authors:** Angelika Zebedin, Friedrich Ladich

**Affiliations:** Department of Behavioural Biology, University of Vienna, Vienna, Austria; National Institutes of Health/NICHD, United States of America

## Abstract

**Background:**

Thorny catfishes exhibit large variations in swim bladder morphology. These organs are of different sizes, forms and may have simple or branched diverticula. The swim bladder plays an important role in otophysans because it enhances their hearing sensitivity by transmitting sound pressure fluctuations via ossicles to the inner ear.

**Methodology/Principal Findings:**

To investigate if a form-function relationship exists, the swim bladder morphology and hearing ability were analyzed in six species. The morphology was quantified by measuring the length, width and height and calculating a standardized swim bladder length (sSBL), which was then used to calculate the relative swim bladder length (rSBL). Hearing was measured using the auditory evoked potential (AEP) recording technique. Two species had simple apple-shaped and four species heart-shaped (cordiform) bladders. One of the latter species had short unbranched diverticula on the terminal margin, two had a secondary bladder and two had many long, branched diverticula. The rSBL differed significantly between most of the species. All species were able to detect frequencies between 70 Hz and 6 kHz, with lowest thresholds found between 0.5 and 1 kHz (60 dB re 1 µPa). Hearing curves were U-shaped except in *Hemidoras morrisi* in which it was ramp-like. Mean hearing thresholds of species possessing smaller rSBLs were slightly lower (maximum 8.5 dB) than those of species having larger rSBLs.

**Conclusions/Significance:**

The current findings reveal a relationship between swim bladder form and its function among thorny catfishes. Relatively smaller swim bladders resulted in relatively better hearing. This is in contrast to a prior inter-familial study on catfishes in which species with large unpaired bladders possessed higher sensitivity at higher frequencies than species having tiny paired and encapsulated bladders.

## Introduction

The swim bladder in fishes plays an important role in buoyancy, in respiration, in the detection of sounds and in sound production [1]–[5]. Due to these different selection pressures the swim bladder morphology varies widely or this organ can be completely reduced. Modifications for sound detection typically involve large swim bladders which are variously connected to the inner ears. In non-otophysines such as clupeids (Clupeiformes), holocentrids (Beryciformes), sciaenids and cichlids (both Perciformes), some or all representatives possess rostral swim bladder extensions which contact the inner ears [6]–[11].

In otophysines, in contrast, swim bladders are connected via a chain of bony ossicles to the inner ear. These Weberian ossicles are found in all representatives of Cypriniformes (carps and minnows), Siluriformes (catfishes), Characiformes (tetras) and Gymnotiformes (South American knifefishes). A complete loss of swim bladders or ossicles has not been reported in any representative of otophysines. Otophysines, which comprise more than 8000 species, evolved an impressive variation in swim bladder and Weberian ossicles morphology, in particular in Siluriformes and to a lesser extent in Cypriniformes [12]–[19]. In catfishes the form of the gas bladder can vary from unpaired apple- or heart-shaped (cordiform) bladders to paired tiny and encapsulated ones to swim bladders with diverticula at the posterior end or over the entire length of the organ. Pseudopimelodids (bumblebee catfish) have large cordiform or tiny gas bladders, which are partly divided into two lateral sacs [20]. Similar modifications were found in ariids (sea catfishes) and auchenipterids (driftwood catfishes) [21]–[22]. Free heart-shaped swim bladders were found in the families malapterurids (electric catfishes), heptapterids (three-barbeled catfishes) and mochokids (squeakers), whereas bony encapsulated ones were found in the families loricariids (armoured catfishes) and callichthyids (callichthyid armoured catfishes) [19]. The swim bladders in doradids (thorny catfishes) are always unpaired and may possess a caudal sac, termed secondary bladder, and numerous diverticula [23]–[24].

Elimination of swim bladders or Weberian ossicles demonstrated that ancillary hearing structures efficiently enhance the hearing sensitivity in otophysines [25]–[31]. Among catfishes, hearing abilities were measured in representatives of 11 out of 36 families: in doradids, pimelodids (long-whiskered catfishes), callichthyids, ariids, pseudopimelodids, malapterurids, heptapterids, mochokids, auchenipterids, silurids (sheatfishes) and ictalurids (North American freshwater catfishes) [19], [32]–[38]. Lechner and Ladich (2008) [19] showed in a comparative investigation on representatives of eight catfish families that species which have large unpaired swim bladders and 3–4 Weberian ossicles hear better above 1 kHz than species which have tiny and encapsulated bladders and only 1–2 ossicles.

Relationships between the morphological variations in accessory hearing structures and hearing abilities were analysed in non-related holocentrids (squirrelfishes), sciaenids (drums or croakers) and in cichlids [6], [8]–[11], [39]–[40]. In general a decrease in the distance between the swim bladder and the inner ear results in an increase in hearing sensitivity at higher frequencies. Besides distance, the size of swim bladders seems to play a role in hearing enhancement. Data on cichlids indicate that species with a large bladder hear better than species with reduced swim bladders [11].

This intrafamilial study was designed to determine if larger swim bladders result in higher auditory sensitivity in thorny catfish (family Doradidae). Thorny catfishes are a potential model to investigate the role of the diversity in swim bladder morphology on hearing within one family. This is because, in contrast to non-otophysines such as cichlids, the factor distance is negligible: bladders are always directly connected to the inner ears via ossicles. Six doradid species were dissected, swim bladder dimensions determined and fish hearing abilities measured.

## Materials and Methods

### Animals

Six species of the catfish family Doradidae were used for this study: *Acanthodoras spinosissimus* (talking catfish) (standard length 55.1–113.2 mm, N = 3), *Agamyxis pectinifrons* (whitebarred catfish) (54.8–59.4 mm, N = 7), *Amblydoras affinis* (53–71.2 mm, N = 7), *Hemidoras morrisi* (71–87.3 mm, n = 9), *Megalodoras uranoscopus* (69.6–122.7 mm, N = 9) and *Oxydoras niger* (ripsaw catfish) (105–168 mm, N = 3). Three specimens of each species were used for morphological investigations and three to nine for hearing measurements.

All fish were purchased from a tropical fish supplier (Transfish, Munich, Germany). Fish were kept in aquaria equipped with sand on the bottom, plants, roots, and various shelters. The tanks were between 70 × 35 × 40 cm (width × height × depth) and 100 × 50 × 50 cm in size. In order to reduce noise within aquaria, water was maintained by external filters. Temperature was kept at 25±1°C and a 12·h:12·h L:D cycle was provided. Fish were fed four to six times per week with frozen chironomid larvae or artificial food.

The study protocol was approved by the Austrian Federal Ministry of Science and Research, permit number GZ 66.006/0023-II/10b/2008.

### Material Examined

Specimen of each species have been deposited in the Naturhistorisches Museum, Wien ( = Vienna) (NMW). *Acanthodoras spinosissimus*: NMW-98200 (2 alc, 62 mm, 112 mm SL), Aquarium purchase. *Agamyxis pectinifrons*: NMW-98201 (1 alc., 62 mm SL) Aquarium purchase. *Amblydoras affinis:* NMW-98202 (5 alc., 62–78 mm SL) Aquarium purchase. *Hemidoras morrisi:* NMW-98203 (1 alc., 128 mm SL) Aquarium purchase. *Megalodoras uranoscopus:* NMW-98204 (1 alc., 185 mm SL) Aquarium purchase. *Oxydoras niger* NMW-98205 (1 alc., 113 mm SL) Aquarium purchase.

### Morphological Measurements

**Figure 1:**
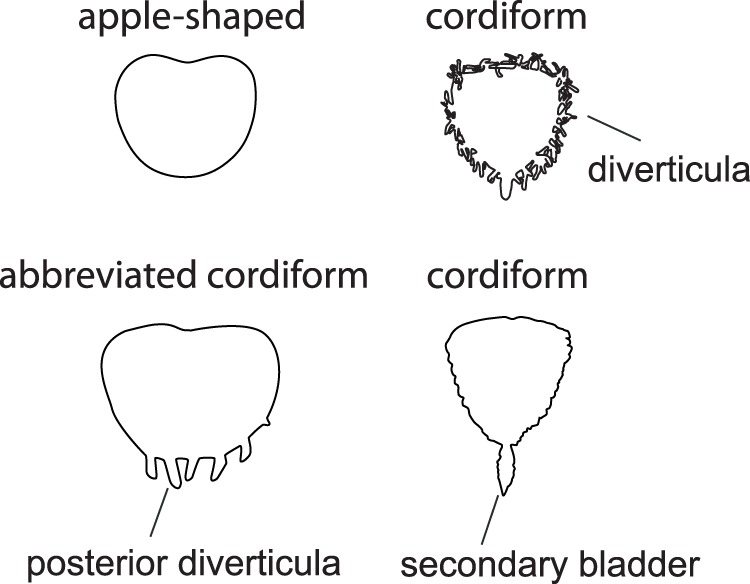
Terminology of shapes and morphological features of doradid swim bladders.

Fish were euthanized using an overdose of tricaine methanesulfonate (MS 222) and were directly fixed in alcohol (70%) for conservation. Dissections were performed under a dissecting microscope (Wild M7). Fish length and swim bladder measures were taken using digital callipers. All measures were taken including the secondary bladders but excluding the diverticula. The swim bladder form was classified following the terminology by [23] (Fig. 1).

Standardized swim bladder length (sSBL) was calculated using the formula sSBL = (l+h+w)/3, where l is length, h is height and w is width according to [19]. The relative swim bladder length (rSBL) was calculated following the formula rSBL = sSBL/SL, where SL is the standard length.

### Hearing Measurements

Hearing sensitivity was measured using the auditory evoked potentials (AEP) recording technique developed by [41] and modified by Wysocki and Ladich [42].

The thorny catfish were mildly immobilized with Flaxedil (gallamine triethiodide; Sigma Aldrich Handels GmbH, Vienna, Austria). The dosage used was 3.07–3.27 µg g^−1^ for *Acanthodoras spinosissimus*, 3.99–5.38 µg g^−1^ for *Agamyxis pectinifrons*, 0.86–3.26 µg g^−1^ for *Amblydoras affinis*, 1.73–2.82 µg g^−1^ for *Hemidoras morrisi*, 3.8–7.01 µg g^−1^ for *Megalodoras uranoscopus,* and 1.73–2.06 µg g^−1^ for *Oxydoras niger.* The lowest dosage that immobilized fish while enabling slight movement of the opercula during the experiments was applied. All auditory measurements were carried out in a bowl-shaped plastic tub (diameter 33 cm, water depth 13 cm, 1 cm layer of gravel) which was lined inside with acoustically absorbent material (air-filled packing wrap) to decrease resonances and reflections. The tub was positioned on an air table (TMC Micro-g 63–540, Technical Manufacturing Corporation, Peabody, MA, USA), which rested on a vibration-isolated plate of concrete. A sound proof chamber, constructed as a Faraday cage (interior dimensions: 3.2 m × 3.2 m × 2.4 m), enclosed the whole setup.

Test subjects were positioned in the centre of the tub, so that the nape of the head was at the water surface. For respiration a pipette was inserted into the fish’s mouth and respiration was effected by a simple, temperature-controlled (25±1°C), gravity-fed water system. The area of the head above the water surface was covered with a small piece of Kimwipes ® tissue paper to keep it moist. Silver wire electrodes (diameter 0.38 mm) were used for recording AEPs. The recording electrode was placed in the midline of the skull over the region of the medulla, the reference electrode cranially between the nares. Both electrodes were pressed firmly against the skin.

Both presentation of sound stimuli and AEP waveform recording were achieved using a modular rack-mount system (Trucker-Davis Technologies (TDT) System 3, Gainesville, FL, USA) controlled by a PC containing a TDT digital signal processing board and running TDT BioSig RP software.

Hearing thresholds were determined for the following frequencies: 0.07, 0.1, 0.3, 0.5, 1, 2, 3, 4, 5 and 6 kHz. Sound stimuli waveforms were created using TDT SigGen RP software. For tone bursts, two speakers (Fostex PM-0.5 Sub and PM-0.5 MKII, Fostex Corporation, Tokyo, Japan) installed 0.5 m above the fish were used. Tone bursts at different frequencies were presented in random order. A hydrophone (Brüel and Kjaer 8101, Naerum, Denmark; frequency range 1 Hz to 80 kHz±2 dB; voltage sensitivity −184 dB re 1 VµPa^−1^) was placed 2 cm from the right side of the animal to determine absolute sound pressure levels (SPLs) under water in the immediate vicinity of the test subject. A second custom-built preamplifier was used to amplify the hydrophone signal (1000×). Sound stimuli consisted of tone bursts played at a repetition rate of 21 s^−1^ and at opposite polarities (90° and 270°). One thousand stimuli of each polarity were presented and the corresponding AEPs averaged by BioSig RP software to eliminate stimulus artefacts. The SPL was reduced in 4 dB steps until the AEP waveform was no longer identifiable. By overlaying replicate traces, the lowest SPL yielding a repeatable AEP trace was determined and regarded as threshold.

### Statistical Analysis

All morphological and physiological data were normally distributed (Kolmogorov-Smirnov-Test). For statistic analysis of the morphological data, a one-way ANOVA was calculated to determine whether rSBLs differ between the species. Differences between the hearing sensitivities of the six species were calculated using a two-way ANOVA followed by a Bonferroni post hoc test. In order to determine whether rSBL affects hearing sensitivities two calculations were carried out. First, the mean thresholds of the three species having smaller rSBLs than 0.165 were calculated for each frequency and compared with those three species having large rSBLs than 0.165 by calculating a two-way ANOVA. Second, Pearson’s correlation coefficient was calculated to correlate the mean hearing thresholds of each specimen at each frequency to rSBL of this species.

All statistical tests were run using PASW 18.0 (SPSS Inc., Chicago, USA).

## Results

### Swim Bladder Morphology

**Figure 2:**
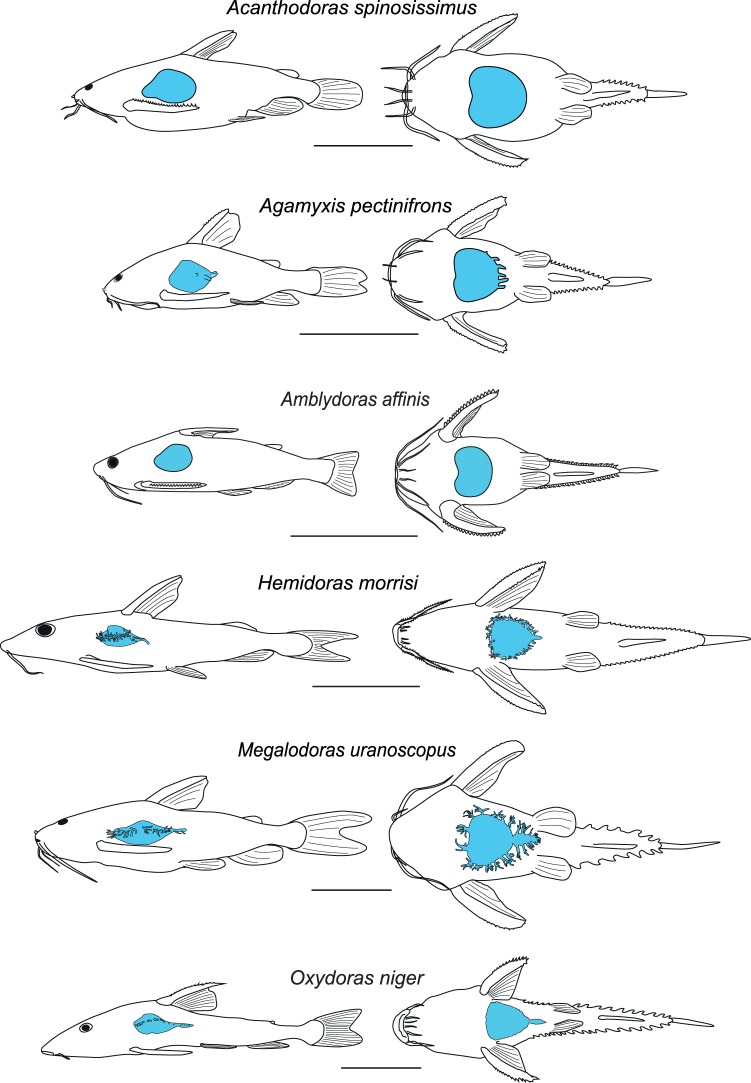
Lateral (left) and ventral (right) view of the six investigated thorny catfish species. Swim bladders are given in blue. Scale bar 3 cm.

The swim bladders of all six species were unpaired and free (not encapsulated). They were classified as being apple-shaped, heart-shaped (cordiform) or abbreviated cordiform (Fig. 1). *Acanthodoras spinosissimus* and *Amblydoras affinis* had apple-shaped swim bladders without any diverticula (Fig. 2). *Agamyxis pectinifrons* possessed abbreviated heart-shaped swim bladders with small, simply formed diverticula on the caudal end of the bladder. These organs were cordiform in *Megalodoras uranoscopus*, *Oxydoras niger* and *Hemidoras morrisi*. In *Megalodoras uranoscopus* and *Hemidoras morrisi* they had many long, branched diverticula anteriorly, laterally and caudally. *Megalodoras uranoscopus* and *Oxydoras niger* had a small secondary swim bladder (Fig. 2).

The rSBL varied between 0.129 in *Hemidoras morrisi* and 0.201 in *Acanthodoras spinosissimus* and differed significantly between species (one-way ANOVA; F_5,17_ = 31.351; p<0.001) (Table 1). Bonferroni post hoc test revealed significant differences in 9 cases (Table 1).

**Table 1:** Swim bladder morphology and measures of species and of swim bladders.

Species	SB morphology	SL (mm)	sSBL (mm)	rSBL
^1)^ *Acanthodoras spinosissimus*	apple-shaped	83.02±15.57	16.64±2.89	0.201±0.003 ^3) 4) 5) 6)^
*^2)^ Agamyxis pectinifrons*	abbreviated cordiform, diverticula	58.15±0.62	11.48±0.28	0.197±0.003 ^3) 4) 6)^
*^3)^ Amblydoras affinis*	apple-shaped	64.83±5.26	10.36±1.05	0.159±0.005 ^4)^
*^4)^ Hemidoras morrisi*	cordiform, diverticula	81.94±2.72	10.50±0.15	0.129±0.006 ^5)^
*^5)^ Megalodoras uranoscopus*	cordiform, secondary bladder, diverticula	112.40±6.13	19.44±0.62	0.174±0.006
*^6)^ Oxydoras niger*	cordiform, secondary bladder	119.06±7.10	17.96±1.64	0.150±0.006

Values: means ± s.e.m. Abbreviations: SB, swim bladder; rSBL, relative swim bladder length; SL, standard length; sSBL, standardized swim bladder length.

Superscripts in the last column indicate significant differences in rSBL between the given species and other species calculated by a Bonferroni Post Hoc test.

### Auditory Sensitivity

**Figure 3:**
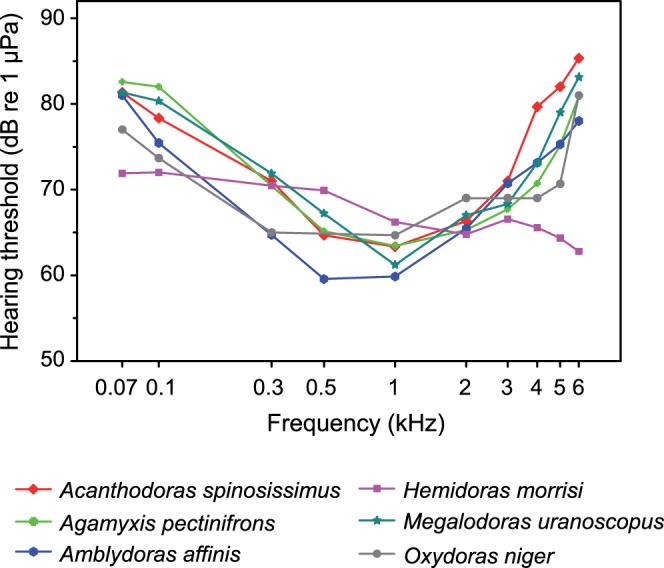
Mean auditory sensitivities of the investigated doradid species.

All species detected tone bursts between 70 Hz and 6 kHz. Hearing curves were typically U-shaped (five out of six species) with best hearing sensitivity located at 0.5 or 1 kHz (Fig. 3, Table 2). *Oxydoras niger* and *Amblydoras affinis* showed lowest thresholds at 0.5 kHz, whereas *Acanthodoras spinosissimus*, *Agamyxis pectinifrons* and *Megalodoras uranoscopus* had best hearing sensitivity at 1 kHz. Hearing thresholds decreased by approximately 15–20 dB from 70 Hz to 0.5/1 kHz and increased at higher frequencies. In contrast, the hearing thresholds of *Hemidoras morrisi* showed an almost constant increase in sensitivity up to 6 kHz. Thus, *Hemidoras morrisi* had better hearing abilities at low and high frequencies compared to the other five species and a lower sensitivity in the mid-frequency range from 0.5–1 kHz. The lowest threshold was found in *Amblydoras affinis* (59.6 dB re 1 µPa at 0.5 kHz) (Fig. 3, Table 2).

**Table 2:** Hearing thresholds (dB re 1 µPa) of thorny catfish species investigated.

F kHz	*Acanthodoras* *spinosissimus*	*Agamyxis pectinifrons*	*Amblydoras affinis*	*Hemidoras morrisi*	*Megalodoras uranoscopus*	*Oxydoras niger*
0.07	81.33±0.33	82.57±0.81	81.00±1.45	71.89±1.21	81.33±0.78	77.00±0.00
0.1	78.33±0.33	82.00±0.58	75.43±0.90	72.00±1.29	80.33±1.87	73.67±0.33
0.3	71.00±2.52	70.43±0.78	64.71±1.11	70.44±0.75	71.89±0.72	65.00±1.53
0.5	64.67±1.33	65.14±1.28	59.57±0.78	69.89±0.61	67.22±1.12	63.67±1.33
1	63.33±0.67	63.43±0.87	59.86±0.88	66.22±1.02	61.22±0.63	64.67±2.73
2	66.33±1.67	65.29±0.68	65.43±1.63	64.78±0.85	67.00±0.33	69.00±1.53
3	71.00±1.00	67.71±1.19	70.71±1.41	66.56±0.69	68.33±0.71	69.00±1.00
4	79.67±1.76	70.71±0.18	73.14±1.98	65.56±0.84	73.11±0.99	69.00±1.53
5	82.00±2.65	75.14±0.55	75.29±1.32	64.33±0.82	79.00±0.94	70.67±2.60
6	85.33±1.87	80.86±0.94	78.00±1.22	62.78±1.00	83.11±0.70.	81.00±1.53

Values: means ± s.e.m. F – frequency.

Comparison of hearing curves revealed a significant difference between most of the species (two-way ANOVA: F_5, 320_ = 50.913, p<0.001) and a significant interaction between species and frequency (F_45, 320_ = 12.476, p<0.001). This indicates that auditory sensitivities showed different trends at different frequencies. *Hemidoras morrisi* differed from all other species in sensitivity.

**Figure 4:**
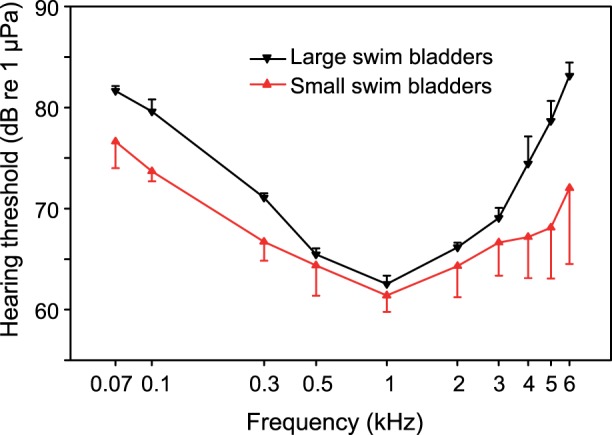
Averaged hearing thresholds of thorny catfish species with large (*Acanthodoras spinosissimus, Agamyxis pectinifrons, Megalodoras uranoscopus)* and small (*Amblydoras affinis*, *Hemidoras morrisi*, *Oxydoras niger*) swim bladders. Standard errors were only drawn in one direction to avoid overlap.

Swim bladders were relatively smaller in *Hemidoras morrisi*, *Oxydoras niger* and *Amblydoras affinis* than in *Acanthodoras spinosissimus*, *Agamyxis pectinifrons* and *Megalodoras uranoscopus* (see rSBL in Table 1). Averaged hearing sensitivity of the three species with smaller swim bladders was significantly higher than in the three species with larger swim bladders (two-way ANOVA: F _1,40_ = 16.245, p<0.001) (Fig. 4). Differences between both groups were not frequency-dependent, indicating a similar trend at all frequencies (two-way ANOVA: F_1,40_ = 0.854, p>0.05) (Fig. 4).

### Correlations between Morphological Structures and Hearing Sensitivities

**Figure 5:**
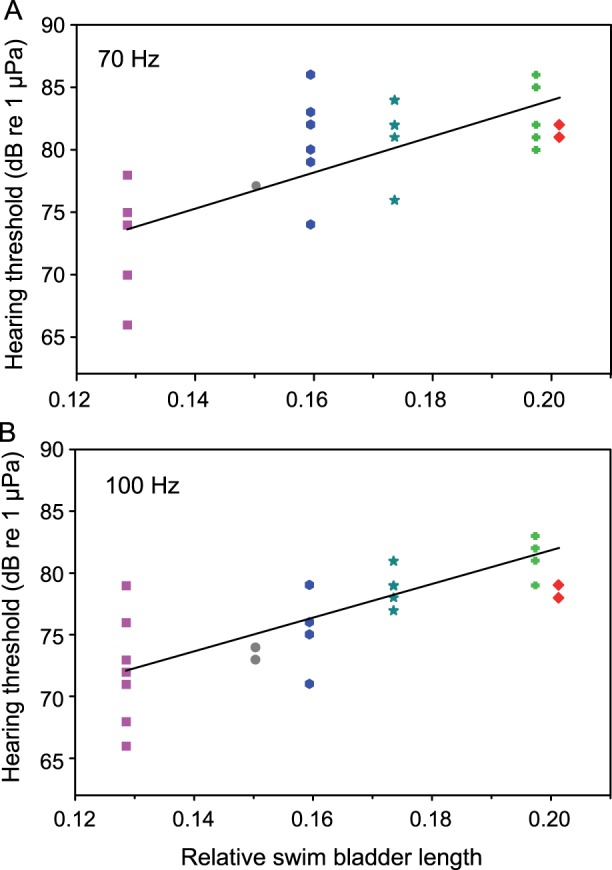
Correlation between mean relative swim bladder length (rSBL) and hearing thresholds at different frequencies. Correlations at A) 70 Hz and B) 100 Hz. Color and symbol code see figure 3. Regression equations: 70 Hz: threshold = 144.7; * rSBL +55.0. 100 Hz: threshold = 136.4 * rSBL +54.5.

**Figure 6:**
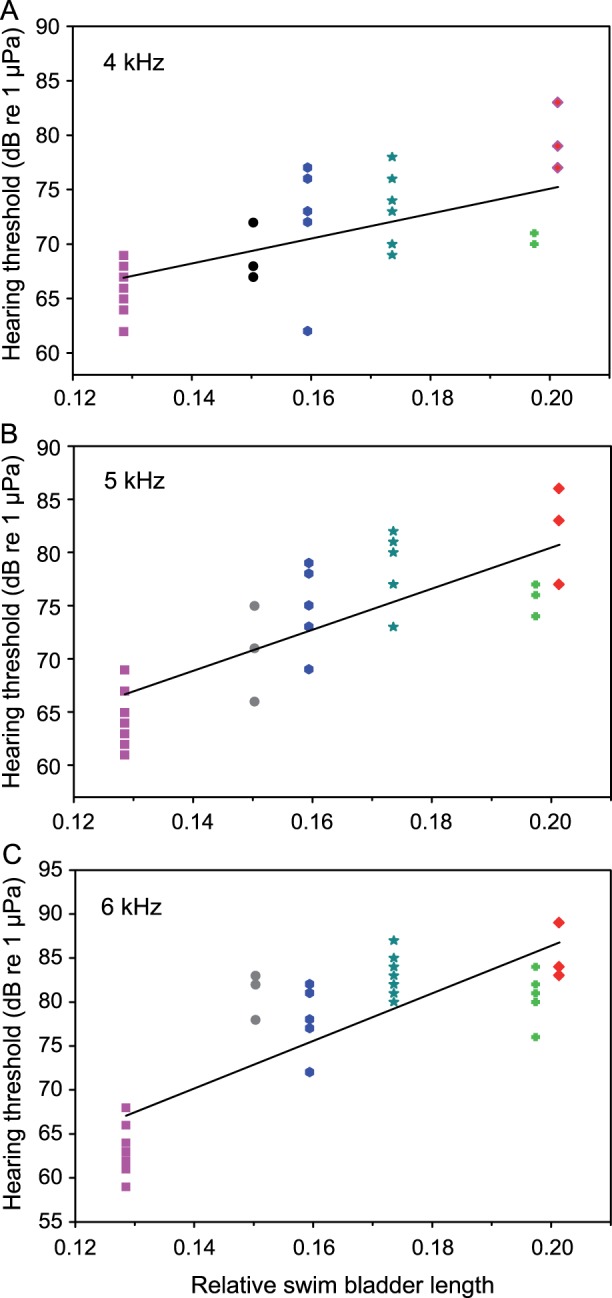
Correlation between mean relative swim bladder length (rSBL) and hearing thresholds at different frequencies. Correlations at A) 4 kHz, B) 5 kHz and C) 6 kHz. Color and symbol code see figure 3. Regression equations: 4 kHz: threshold = 114.3 * rSBL +52.2; 5 kHz: threshold = 193.3 * rSBL +41.9; 6 kHz: threshold = 270.4 * rSBL +32.3.

The rSBL was positively correlated to the hearing thresholds at 70 and 100 Hz (Pearson’s correlation: 70 Hz: r = 0.75, p<0.001; 100 Hz: r = 0.69, p<0.001, N = 38) (Fig. 5A, B) and at 4, 5 and 6 kHz (Pearson’s correlation: 4 kHz: r = 0.60, p<0.001; 5 kHz: r = 0.76, p<0.001; 6 kHz: r = 0.81, p<0.001, N = 38) (Fig. 6 A, B, C). Species with larger swim bladders had higher thresholds in five out of ten frequencies. No significant relationship between relative swim bladder size and hearing were found between 0.3 and 3 kHz.

## Discussion

### Swim Bladder Morphology in Catfishes

Thorny catfish comprise 92 species in 31 genera [43], [44] and exhibit the largest variety of swim bladder modifications among catfishes [23]. The swim bladder morphology described in the present study mostly resembles that described by [23]. The swim bladder of *Amblydoras affinis* is similar to that of *Amblydoras bolivarensis* (apple-shaped with completely smooth walls) [23]. Another apple-shaped bladder was found in *Acanthodoras spinosissimus*, which is comparable to the organ of *Acanthodoras cataphractus*. The swim bladder of *Megalodora*s *uranoscopus* from the current study resembles that of *M*. *uranoscopus* and *Megalodoras guayoenis*, i.e. cordiform with a secondary bladder and many well-developed and often branched diverticula. Birindelli et al. (2009) [23] showed that small specimens of *Megalodoras uranoscopus* possess diverticula all over the swim bladder, whereas diverticula are lacking anteriorly in larger specimens. The size of the secondary chamber and the number of diverticula increase with growth. *Hemidoras morrisi* exhibited a cordiform swim bladder with many branched diverticula. Birindelli et al. (2009) [23] mentioned that these diverticula became thinner and more branched in larger specimens of *Hemidoras* species. In *Agamyxis pectinifrons* there were three to five small posterolateral diverticula, whereas in *Agamyxis albomaculatus* there was only a single posterolateral diverticulum on either side of the terminal diverticulum according to [23]. Kaatz and Stewart (2012) [24] described the swim bladder morphology in this family similar to [23], except that all extensions including secondary bladders were termed diverticula. Kaatz and Stewart (2012) [24] provided absolute swim bladder measures. Swim bladders varied from 0.4 to 4.5 cm in length, from 0.5 to 3.3 cm in width and from 0.2 to 2.0 cm in depth. The number and lengths of diverticula were species-specific and varied from shorter diverticula less than a few mm in length (e.g. *Agamyxis pectinifrons*) to longer diverticula about 1/3 of the swim bladder length (e.g. *Oxydoras niger*) [24].

Recent studies on swim bladder morphology in other catfish families revealed further differences in gross morphology, but these intrafamilial differences were smaller than in thorny catfishes [21]–[22]. Gas bladders in auchenipterids, which are closely related to doradids [45]–[46], varied to a lesser extent than in doradids, but more than in other catfish families such as pseudopimelodids and ariids [20]–[22], [45]. Auchenipterids possessed either cordiform swim bladders with smooth walls (*Glanidium, Pseudauchenipterus*), almost apple-shaped bladders (*Centromochlus* spp.) or bladders reduced in size and partially or completely ossified (*Ageneiosus inermis*). *Tocantinsia piresi* and *Asterophysus batrachus* had distinctive-looking bladders with either two or many diverticula. Variations were also found within genera: *Trachycorystes menezesi* had a simple cordiform organ, whereas *Trachycorystes trachycoryst*es had a pair of lateral diverticula and a well-developed terminal diverticulum. The bladder of the genus *Auchenipterichthys* was characterized by having a secondary bladder [22]. Birindelli et al. (2012) [22] discussed that such morphological variation may be related to the elastic spring apparatus. The diversity of swim bladder shapes in ariids is moderate compared to the high diversity in doradids. In ariids most species have a cordiform bladder with smooth external walls (*Bagre*); a few species have well-developed secondary bladders (*Sciades* spp.) and some have apple-shaped bladders (*Galeichthys ater*, *G. feliceps* and *G. peruvianus*) [21]. Gas bladders either completely lack diverticula or possess rounded bulges or blister-like swellings (e. g. *Aspistor quadriscutis*). In contrast to the diversity in the swim bladder form in thorny catfishes, these organs in pseudopimelodids varied only minimally. The organ in pseudopimelodids is large and cordiform (*Cephalosilurus albomarginatus*, *Batrochoglanis*) or moderately sized and apple-shaped (*Lophiosilurus alexandri*). Representatives of the genera *Pseudopimelodus* and *Cruciglanis* have diminutive organs partially divided into two lateral sacs, where the parapophyses of the fourth vertebra partially cover the bladder anteroventrally [20].

The relative size of swim bladders varied between different species of thorny catfishes. The largest rSBl was found in *Acanthodoras spinosissimus* (0.201), the smallest in *Hemidoras morrisi.* (0.129). Quantitative data on relative swim bladder lengths are found only in [19]: Kaatz and Stewart (2012) [24] did not calculate ratios between swim bladder measures and the (standard) length of fish. Lechner and Ladich (2008) [19] reported that the rSBL of free unpaired swim bladders varied from 0.084 in *Malapterurus beninensis* (Malapteruridae) to 0.152 in *Synodontis schoutedeni* (Mochokidae). In catfish species with tiny paired and encapsulated bladders, the rSBL was much smaller and varied between 0.016 in *Hemiodontichthys acipenserinus* and 0.057 in *Ancistrus ranunculus* (Loricariidae) [19]. This comparison reveals that free unpaired bladders (Ariidae, Pseudopimelodidae, Malapteruridae, Heptapteridae, Mochokidae, Auchenipteridae and Doradidae) are larger than paired bladders (Loricariidae and Callichthyidae). Intrafamilial variation among doradids in rSBL (1∶1.6) is much smaller than the interfamilial variation (1∶9.5) described by [19]. Siluriformes comprise numerous buttom-dwelling fishes; thus, most species do not require large swim bladders for buoyancy. Nevertheless, catfishes and all other otophysines never completely lose their swim bladders, in contrast to other fish taxa (e.g. gobiids, bleniids, cottids), most likely because of their auditory function [14], [46].

### Hearing Sensitivity in Catfishes

Overall hearing abilities of the investigated doradids differed between most species, but showed similar general characteristics. The hearing curves where U-shaped with best sensitivities between 0.5 and 1 kHz in five out of six species The hearing curve of *Hemidoras morrisi* differed from all other species because of its ramp-like shape with an almost constant decline towards higher frequencies. The U-shaped hearing curves of *Acanthodoras spinosissimus*, *Agamyxis pectinifrons*, *Amblydoras affinis*, *Megalodoras uranoscopus* and *Oxydoras niger* resemble the audiogram previously gained in the striped Raphael catfish *Platydoras armatulus* (formerly *P. costatus*), another representative of thorny catfishes [34], [47]. Surprisingly, the hearing threshold of *Agamyxis pectinifrons* was 10–30 dB higher in the previous than in the current study [34]. Because the AEP protocols utilized in both studies were similar, we assume that fish size is responsible for this difference in sensitivity. The fish in the prior study covered a smaller size range (2.1–7.9 g) than in the present one (6.9–9.0 g). Accordingly, sensitivity probably improved during growth.

Differences in hearing sensitivities were also found in other catfish families such as callichthyids and loricariids. Lechner and Ladich [19] showed that within callichthyids *Corydoras sodalis* had higher hearing thresholds (approx. 7 dB at all frequencies tested) than *Dianema urostriatum*, which resembles difference between *Acanthodoras spinossisimus* and *Amblydoras affinis* in the current study. Similar differences were found among loricariids, where *Ancistrus ranunculus* had higher thresholds than *Hemiodontichthys acipenserinus* and *Hypoptopoma thoracatum* [19]. No significant difference was found among pimelodids [34]. This may be due to the fact that *Pimelodus pictus* and *P. blochii* belong to the same genus.

Comparing different families with regard to hearing abilities reveals a general trend, namely that relative swim bladder size affects hearing sensitivities. Ladich (1999) [34] determined that callichthyids had lower auditory sensitivities than pimelodids (*Pimelodus blochii* and *P. pictus)* and doradids (*Platydoras armatulus*), whereas there was no difference between the latter two families. Lechner and Ladich (2008) [19] observed that loricariids and callichthyids had lower sensitivities above 1 kHz than ariids, pseudopimelodids, malapterurids, heptapterids, mochokids and auchenipterids.

### Relationship between Swim Bladder Morphology and Hearing Sensitivity

The present study investigated whether the variation in swim bladder morphology affects hearing sensitivity in thorny catfishes. Regarding their rSBLs the doradids were divided into two groups: fish with larger (*Acanthodoras spinosissimus*, *Agamyxis pectinifrons* and *Megalodoras uranoscopus*) and fish with smaller swim bladders (*Hemidoras morrisi, Oxydoras niger*, *Amblydoras affinis*). Surprisingly, fish with smaller swim bladders had slightly better hearing abilities. This finding is in contrast to former studies. Lechner and Ladich (2008) [19] described pronounced differences in rSBL between catfishes possessing large unpaired (mean rSBL of 0.121) and those having small paired bladders (mean rSBL of 0.037). These differences resulted in significantly better hearing abilities at frequencies above 1 kHz, with mean differences of about 5 dB at 1 kHz until up to more than 20 dB at 5 kHz [19]. We expected smaller differences in hearing sensitivities because differences in rSBLs between thorny catfishes having larger and smaller rSBLs was smaller (mean rSBLs: 0.191 versus 0.146) than between different catfish families (0.015 versus 0.152). Nevertheless, a comparison of hearing sensitivities of the thorny catfish species with smaller and larger rSBLs yielded unexpected results. Clearly, factors other than swim bladder size affected hearing. Did swim bladder diverticula improve hearing sensitivities? This was not the case: *Amblydoras affinis*, with its simple apple-shaped swim bladder without any diverticula had the lowest auditory threshold, whereas species with a cordiform gas bladder and diverticula had poorer sensitivities. None of the swim bladders in this study were covered by bones, enabling them to vibrate freely. The differences in hearing abilities may be explainable by differences in accessory hearing structures (swim bladder, Weberian ossicles), by inner ear morphology or by ontogenetic development [19], [48].

The effects of different swim bladder size on hearing have not been studied in fishes except in catfishes. Typically, investigators concentrated on the distance between swim bladder (including anterior extensions) and inner ear. Smaller distances positively affect the hearing sensitivities in holocentrids and to some degree in sciaenids. The Hawaiian squirrelfish *Myripristis kuntee* showed lower auditory thresholds and detected a wider frequency range than *Adioryx xantherythrus* [6], [8]. *Myripristis kuntee* possesses anterior swim bladder horns, which directly contact the ear, whereas the distance between bladder and inner ear in *Adioryx xantherythrus* is significant larger. The situation is more complicated in sciaenids. No clear differences in absolute hearing threshold were observed in sciaenid fishes in subsequent studies. The weakfish *Cynoscion regalis* detects frequencies up to 2000 Hz, the spot *Leiostomus xanthurus* frequencies only up to 700 Hz. The hearing differences were explained by different swim bladder-inner ear configurations. In weakfish the bladder has a pair of anterior horns and terminates close to the ear, while in the spot it terminates farther away from the ear [9], [49]. In contrast, Horodysky et al. (2008) [50] showed that among sciaenids, *Menticirrhus saxatilis* – which lacks swim bladders as adults – exhibits best hearing sensitivities below 600 Hz. They also showed that thresholds of species with anterior extensions of their swim bladders (e.g. Atlantic croaker, spotted seatrout) were not significantly lower than those of fishes lacking these projections (e.g. northern kingfish, red drum) [50]. In cichlids, anterior swim bladder extensions improve hearing sensitivities above 300 Hz in the orange chromide *Etroplus maculatus* and in *Paratilapia polleni* [11]. Swim bladder size seems to be important (besides distance to the inner ear). *Hemichromis guttatus*, which lacks anterior extensions but possesses a large swim bladder, has an auditory sensitivity similar to *Etroplus maculatus* and *Paratilapia polleni* up to 3 kHz. In contrast, *Steatocranus tinanti*, which has a tiny swim bladder, detects sounds frequencies only up to 700 Hz.

### Conclusion

The present investigation showed that thorny catfish with smaller swim bladders had slightly better hearing abilities than species with larger ones. Although this result is unexpected underlines that the relationship between swim bladder morphology and auditory sensitivity is not always straightforward: larger swim bladders and shorter distances between swim bladders and inners ear result in improved hearing. Horodysky et al. (2008) [50] showed that, among sciaenids, swim bladder reduction and the presence or absence of anterior extensions do not affect hearing sensitivity. Other factors such as the surrounding of the bladder (bony encapsulation) and thus its vibrations patterns, the fine structure of the swim bladder wall, morphological differences in the Weberian ossicles, or the inner ear morphology might influence hearing sensitivity in fishes in general and in thorny catfishes in particular.
